# Do Polarization Narratives Apply to Politics on the Periphery? The Case of Atlantic Canada

**DOI:** 10.3389/fsoc.2021.655880

**Published:** 2021-10-22

**Authors:** Rachel McLay, Howard Ramos

**Affiliations:** ^1^ Department of Sociology and Social Anthropology, Dalhousie University, Halifax, NS, Canada; ^2^ Department of Sociology, Western University, London, ON, Canada

**Keywords:** political polarization, Atlantic Canada, public opinion, regional politics, immigration

## Abstract

Researchers, policymakers, and the public often claim that “extreme” political views have become increasingly commonplace and that polarization on issues of race and immigration has become a central dilemma for contemporary politics. The popular narrative of political polarization captures tensions that many are noticing and experiencing. However, there is also significant confusion around the concept, as well as gaps between popular perceptions and empirical findings on the different forms of polarization and their prevalence across regions. It is unclear to what extent polarization describes a global phenomenon, as its national and subnational manifestations vary considerably, produced from distinct local histories as well as diffuse transnational forces. While the United States is often treated as ground zero for political polarization, nearby Canada does not appear to be experiencing polarization to nearly the same degree. Using data from a 2019 survey on Atlantic Canadians’ political views and perceptions of change, this paper examines whether underlying forms of political polarization are manifesting in the region. We assess whether mass ideological polarization and partisan sorting can be found in Atlantic Canada, looking at socio-cultural and economic dimensions of political values. We also examine perceptions of polarization in the region, using Multiple Correspondence Analysis to observe underlying associations between perceptions, extreme or polarized views, and partisanship. This mapping approach provides insight into latent patterns often missed by more traditional methods.

## Introduction

Researchers, policymakers, and the public all frequently lament that “extreme” political views have become increasingly commonplace (Halikiopoulou, 2018; Moss and O’Connor, 2020) and that polarization, especially on issues of race and immigration, has become a central dilemma for contemporary politics. Research links increased polarization to regional, cultural, and economic divides (Inglehart and Norris, 2016; Scala and Johnson, 2017), and finds that it is aggravated by toxic new media landscapes (Bail et al., 2018; Dahlgren, 2018; Hong and Kim, 2016; c.f.; Barberá, 2015). However, it is often unclear to what extent polarization describes a global phenomenon, or whether it is a pattern observable mainly in in several key cases among prominent states. National and subnational manifestations of polarization vary considerably, as they are produced from distinct local histories as well as diffuse transnational forces. For example, while the U.S. is often treated as ground zero for political polarization, nearby Canada does not appear to be experiencing polarization to nearly the same degree.

Still, narratives of polarization and division—especially via the rise of right-wing populism—are present in the English Canadian consciousness, cropping up regularly as a boogeyman in news media (see, e.g., Nuttall, 2017; Berthiaume, 2019; Warnica, 2019). There is a persistent fear that, if the U.S. sneezes, Canada will catch a cold. Ever since the election of Donald Trump in 2016, the country has been closely self-monitoring for symptoms of division, polarization, and anger in the political sphere. Attempts to import far-right politics into Canadian electoral politics have largely failed thus far. Nevertheless, scholars have raised concerns about the presence of right-wing extremism in Canada (see, e.g., Parent and Ellis, 2014; Perry and Scrivens, 2019), and other Canadian studies have attributed polarization to factors such as regional differences (Walks, 2006) and social media (Gruzd and Roy, 2014). In this paper, we consider how different forms of political polarization, including perceived polarization, operate in Atlantic Canada, using data from a 2019 survey on residents’ political views and perceptions of change. The case has the potential to provide new insights into the role of polarization narratives on the “periphery,” and it provides an important counterexample to common arguments made about the political attitudes endemic in rural, White locales in North America (see, e.g., Cramer, 2016).

In these understudied, peripheral Canadian provinces, we examine whether signs of polarization can be found in the public’s political attitudes and perceptions to see if patterns there mirror patterns observed in the United States. We begin by providing an overview of the broader literature on forms of political polarization. We then consider whether these dynamics are present in the Atlantic Canadian context. More specifically, we look for evidence of mass ideological polarization on socio-cultural and economic issues. Next, we consider the extent to which partisan sorting and partisan polarization are occurring, sharpening divisions between party supporters. Finally, we assess how people are perceiving polarization, including who is observing it most frequently and whether such observations are clustering with more extreme or polarized viewpoints and with partisanship.

### Forms of Polarization

Polarization is often treated as a straightforward or self-explanatory concept in its popular usage. However, the term typically stands for a complex mix of several distinct analytical forms: mass ideological polarization, elite polarization, partisan sorting or partisan polarization, affective polarization, and perceived polarization. The popular understanding typically focuses on mass ideological polarization, in which the public is imagined to be divided into two camps, holding views at opposite ends of the political spectrum. This bimodal distribution of views with clustering at the poles is “polarization” in the most technical sense of the term (Fiorina and Abrams, 2008). However, such a pattern is highly unlikely to be seen in practice. On certain issues, the public has become more divided; (Dunlap et al., 2016), or their views have become more widely dispersed over time (Adams, 1997). But, across the plurality of issues at stake in the political sphere, only a minority of individuals hold views that consistently place them at one end of an ideological spectrum (Cochrane, 2015).

Although broad ideological polarization is rare within the general public, polarization among political elites has been much better supported by scholarly research. Elite polarization is evidenced by increasing homogeneity in the political positions of party elites, as their differences on a variety of political issues are reduced to a single liberal-conservative dimension (Carmines et al., 2012; Hare and Poole, 2014). In the U.S., for example, the positions of Democrat and Republican politicians on welfare and taxes are likely to be strongly correlated with their views on immigration and LGBTQ + rights. But, among the general population, researchers often find multiple distinct dimensions, most notably the socio-cultural and economic dimensions (Carmines et al., 2012; Hare and Poole, 2014). Within these dimensions, the left and right wings each carry “family resemblances” on related issues and questions (Cochrane, 2015), but they do not form homogeneous clusters. In short, there is great diversity among people’s views within and across dimensions, as well as many areas of overlap between those on the left and right.

Nevertheless, partisan sorting can often be seen on specific issues. Partisan sorting is the process by which individuals with similar views cluster into parties over time due to their shared positions on valued issues (Kevins and Soroka, 2018). For example, in the case of abortion, partisan differences in the electorate emerged about 10 years after party platforms diverged (Adams, 1997). As party positions and elite debates become more entrenched, partisans either change their party affiliation to align it with their views or attenuate their views to better align them with their party affiliation (Fiorina and Abrams, 2008). In this way, elite polarization contributes to perceptions of broader ideological polarization, as partisans take cues from political leaders and party activists and sort themselves accordingly. However, resulting divisions are largely caused by “people changing their parties instead of their attitudes” (Adams, 1997, p. 729; see also Hare and Poole, 2014). Individuals usually retain mixed views, but they align with parties that reflect their views on whichever issues they prioritize most highly (Cochrane, 2015). However, there is evidence that some degree of partisan polarization is occurring, beyond partisan sorting. The views of party supporters have gradually pulled away from the center, even if they have not yet resulted in polarized extremes. For example, Democrats and Republicans in the U.S. have become less centrist over time (Levendusky, 2009; Abramowitz and Saunders, 2008).

Given the limited evidence of large-scale ideological polarization occurring in the general public, many researchers in the last several years have turned their attention to affective polarization. This form of polarization considers how partisans feel about their political allies and rivals (see Iyengar et al., 2012; Iyengar and Westwood, 2015; Rogowski and Sutherland, 2016; Druckman and Levendusky, 2019; Iyengar et al., 2019; West and Iyengar, 2020; Wilson et al., 2020; Arbatli and Rosenberg, 2021). Sometimes allies and rivals are defined by party-based identities; for example, U.S. Republicans may have increasingly positive feelings toward other Republicans and increasingly negative feelings toward Democrats. More general left- or right-wing identities can also produce the affective allegiances that define this form of polarization (Mason, 2018). Affective polarization can include changes in the tone and tenor of discourse, as well as increased anger or incivility in political conversation, and the forming of in-groups and out-groups based on political views or alignments. Mason (2018) finds that these identity-based antagonisms are independent of actual policy differences. Consequently, polarization should be understood as more than simply the “distance” between opposing views (DiMaggio et al., 1996). It must also include the irreconcilability of differing parties or political identities. If the heightened presence of anger and antagonism in political discourse signals more entrenched, uncompromising political positions or group alignments, then measures of polarization that ignore changes in discursive tone are likely to underestimate the extent of existing divisions.

It is all these forms of polarization, rather than the presence of large-scale ideological polarization, that have contributed to a political climate that “undeniably feels different” (Hetherington and Weiler, 2009). Increases in elite and affective polarization over the last few decades are commonly mistaken for or conflated with mass ideological polarization, resulting in higher levels of perceived polarization (see, e.g., DiMaggio et al., 1996; Westfall et al., 2015; Vegetti, 2019; Wilson et al., 2020). Media have played a key role in amplifying narratives of division, increasing affective divides and perceptions of polarization (DiMaggio et al., 1996; Fiorina and Abrams, 2008; Wilson et al., 2020). Such perceptions are further compounded by social media and other online spaces, which serve as “filter bubbles” or “echo chambers,” increasing the ideological gulf between opposing viewpoints or drawing more attention to existing ideological differences (Baldassari and Bearman, 2007; Levendusky and Malhatra, 2016; Yang et al., 2016; Wilson et al., 2020).

While perceptions of heightened polarization predate social media, such platforms have been instrumental in shaping current manifestations of it. Social media CEOs Mark Zuckerberg and Jack Dorsey have both publicly acknowledged the roles their respective platforms, Facebook and Twitter, have played in fomenting political divides, and both have expressed the goal of reducing this polarization. But the very nature of social media as many-to-many personal communication aggravates polarization and makes it more visible, as individuals put their identities—including political identities and partisan affiliations—on display (Iyengar and Westwood, 2015). Baldassari and Bearman (2007), for example, find that people generally overestimate ideological similarity among their close associates, but “takeoff issues,” which gain attention and generate discussion, can disrupt stable relationships by alerting people to previously unknown or unacknowledged differences. Social media increases the expression of these differences, making them harder to ignore. Moreover, social media tends to amplify polarizing content (Wilson et al., 2020), and it is frequently used by politicians and activists to share messages with the public that trigger highly polarized responses, increasing perceptions of mass ideological polarization. These messages increasingly defy geographical boundaries (see, e.g., HerdaĞdelen et al., 2013). As a result, via social media, polarizing politics diffuse beyond the individual political contexts that stoke them, potentially creating a more global phenomenon—or the perception of one. The polarization narratives dominating the U.S., for instance, are shared with and absorbed by Canadians. These narratives are likely to have an effect, regardless of differences in the countries’ historical and political contexts.

### How Could Polarization Manifest in Atlantic Canada?

Research on Canada’s distinct political history and multi-party system often highlights the country’s uniqueness (Lipset, 1990; Adams, 2003; Johnston, 2017). Not only has it resisted the pull towards two main parties seen in similar institutional contexts, but, for many years, it was dominated by a centrist party, the Liberals (Johnston, 2017). In this way, the simple left/right divide has not always been especially meaningful in the Canadian context (Cochrane, 2015). Canadians’ political attitudes have historically been characterized as more pragmatic and less dogmatic than those of Americans (Gibbens and Nevitte, 1985). In such a context, polarization seems unlikely to take root. Nevertheless, Canada faces many cultural forces similar or analogous to those facing the U.S. For example, its history of colonization and regional differences contributes to divisions like those which have fueled polarized debates in other regions. Notably, Canada faces attitudinal tensions and divisions around reconciliation and addressing its historical and ongoing colonial relationships with Indigenous peoples (Wilkes, 2004; Ramos, 2008). The potential for polarization in Canadian politics clearly exists, especially on social identity issues. In addition to problems of systemic racism and debates over the admission of racialized immigrants and refugees, regional differences are highly salient in Canada (Cairns, 1968; Banting and Soroka, 2020).

However, much of the work that has analyzed the character of provincial or regional politics has focused on either the Western provinces, with attention to the ultra-conservative movement and populist politics in the region (Harrison, 2000; Wiseman, 2011; Banack, 2013; Sayers and Stewart, 2013) or Quebec, with a focus on its distinct political culture and the challenge this poses to Canadian unity (Nadeau and Belanger, 2012; Laxer, 2019; Turgeon et al., 2019; see also Johnston, 2019; Banting and Soroka, 2020). Atlantic Canada, meanwhile, has largely been ignored. The region is an interesting case study for the spread of polarization and “extreme” views. Although it is predominantly rural and economically depressed—prompting researchers to draw parallels with the U.S. “rust belt” (Kaida et al., 2020)—the Atlantic region has not thus far been identified as a major site of far-right organizing or anti-immigrant sentiment (Perry and Scrivens, 2019). In fact, despite its reputation for being averse to change, polls over the last several years consistently find that Atlantic Canada has the most progressive views on immigration and socio-cultural diversity in Canada (e.g., EKOS Politics, 2019; Gunn, 2019), defying stereotypes formed by findings in other white, rural, and economically depressed regions in North America (see, e.g., Cramer, 2016). Probing the political views of Atlantic Canadians may reveal a great deal about the role of local histories and regional characteristics, the effects of exogenous shocks from nearby political fields, and the ways in which new linkages between distant fields, enabled by online social spaces, can interact with regional histories to spur change, potentially diffusing forms of polarization in the region.

Existing research certainly points to signs of increasing polarization in Canada overall. Since the 1980s, elite polarization has increased in the country, as political parties have more clearly carved out spaces on the left and right (Cochrane, 2015). There is evidence that Canadian politics may be experiencing a “flattening” of multiple political dimensions onto a single liberal-conservative dimension, similar to the bifurcation observed in two-party systems like the U.S. (see Hare and Poole, 2014). Cochrane (2010) finds that, on the political left, more coherent “bundles” of views are forming, spanning both socio-cultural and economic issues (see also Kevins and Soroka, 2018); a guiding principle, equality, can influence both kinds of opinions. Therefore, holding equality as a core political value can yield a high degree of consistency across issues. On the right, there is less ideological coherence across socio-cultural and economic views, as a result of the distinct strains of conservatism in Canada and their separate traditions, beliefs, and moral foundations (see Wiseman, 2011; Banack, 2013). Electorally at the federal level, however, these strains officially merged in 2003 to form the Conservative Party, successfully “uniting the right” after years of fluctuation. Thus, while multiple dimensions are still prevalent on the right, strong alliances have formed across them.

Signs of polarization among political leaders and parties, however, do not necessitate a similar degree of polarization within the general public. But elite polarization stimulates partisan sorting, may also lead to partisan polarization, and can increase affective and perceived polarization within the public. For example, Johnston (2019) finds increasing affective polarization and negative partisanship in Canada, also since the 1980s. In multi-party systems, it is possible for negative partisanship to have multiple targets (McGregor et al., 2015). Nevertheless, in Canada, there is a substantial and growing affective distance between two groups: the Conservatives and “everybody else” (Johnston, 2019; see also McGregor et al., 2015). This suggests that despite internal differences, Conservatives are affectively linked by a shared Conservative identity, while antipathies between Conservatives and those with liberal or left-leaning views, regardless of their party affiliation, have grown substantially, comparable to the increasing hostilities between U.S. Republicans and Democrats (Johnston, 2019). While these findings point to patterns of polarization among partisans in Canada, they do not necessarily apply to the broader public. Only a small proportion of the Canadian population belongs to a political party, and changes in party support are common (LeDuc et al., 1984; Cross and Young, 2004; Cochrane, 2014). It seems unlikely, therefore, that a single dimension can capture the politics of the Canadian public; multiplicity is the rule, while consistency remains the exception.

Nevertheless, the fear that intense polarization will come to define Canadian politics remains pervasive. The rise of social media and online news are likely to aggravate these fears and perceptions. Social media may increase perceptions of polarization in Canada by increasing affective polarization; research has shown that more “polarized” individuals perceive higher levels of polarization (Van Boven et al., 2012; Westfall et al., 2015). New media may also facilitate political information-gathering and network-building across multiple geographies, which can increase Canadians’ exposure to and participation in transnational movements (see Wood, 2015). For example, movements like Occupy Wall Street, Black Lives Matter, and #MeToo originated in the U.S. but were quickly echoed in Canada and represented in collective action across the country and around the world. While parallel movements often attempt to localize messages, the global justice framing that is commonly used by movements tends to emphasize similarities across contexts rather than national, regional, or local distinctiveness. Therefore, it is possible that forms of polarization may begin to take root in unlikely places across the globe, despite many historical, contextual differences. In the rest of the paper, we explore this possibility by looking at patterns in Atlantic Canadians’ views, partisanship, and perceptions in order to better understand whether forms of polarization can be found in this peripheral region.

## Methods

To assess whether polarization can be found in the Atlantic Canadian context, we first consider whether there is evidence of ideological polarization on economic and socio-cultural issues within the general population in Atlantic Canada. We also consider whether views on socio-cultural issues are correlated with those on economic issues, indicating the presence of substantial clustering across key dimensions of Atlantic Canadians’ politics. Next, we examine the views of party supporters to determine whether there is evidence of partisan sorting, represented by fairly distinct clusters of views among people who support each of the prominent political parties in Canada. If there is such clustering, it would provide evidence of partisan sorting. Among those who do not support a specific party, we examine vote choice to see if clustering emerges around these views absent self-declared party affiliations. We also consider perceived polarization by examining the extent to which people perceive increases in polarization, both ideological and affective in nature. To better understand these perceptions of polarization and their relationship to more “extreme” or polarized views on socio-cultural and economic issues, we map how these perceptions are linked to political views, partisanship, and the use of social media.

Our analysis draws upon survey data collected through a telephone survey of 1,072 Atlantic Canadians, which asked participants about their views on political and social issues, as well as open-ended questions about their perceptions of changes in Canadian politics. The survey was conducted between January and March 2019. Participants were recruited through a random selection of telephone numbers assigned to the region; 77.4% were landlines and 22.6% were mobile phones. Only residents of Atlantic Canada who were 18 years or older when contacted were invited to participate. Most surveys were completed in English, but 4% were completed in French. By province, 49.1% of participants resided in Nova Scotia (NS), while 29.1% lived in New Brunswick (NB), 15.1% in Newfoundland and Labrador (NL), and 6.7% in Prince Edward Island (PE). The overall population breakdown of Atlantic Canada in 2020 includes 40.1% in NS, 32.0% in NB, 21.3% in NL, and 6.5% in PE.

### Measuring and Mapping Polarization

If ideological or partisan polarization are present in Atlantic Canada, they should be observable either as the bipolar clustering of views or as the clustering and sorting of the general population according to political affiliation. To examine ideological differences, we analyze people’s views on socio-cultural and economic issues. Our survey asked participants to rate their agreement with twelve different statements on a scale from 1 to 5, with 1 meaning “strongly disagree” and 5 meaning “strongly agree” (see Table 1A in the Supplementary Appendix). To get an overall picture of participants’ views, we began our analysis by determining if these separate measures could be collapsed into two scores, representing participants’ views on socio-cultural and economic issues using a scale from very conservative to very progressive. Most of the statements were worded favouring a progressive stance: that is, agreement signified a liberal or progressive view, while disagreement signified a more conservative view. For statements where the opposite was true, we inverted participants’ responses to match the scaling of other questions.

To determine whether the statements were suitable for combining into scores, we used Cronbach’s alpha to measure scale reliability and exploratory factor analysis to assess the unidimensionality of items. Using this approach, we determined that there is a main latent factor in the set of statements on socio-cultural issues, which signals a degree of ideological consistency between responses. The Cronbach scale reliability coefficient was 0.82 for these statements, and the overall Kaiser-Meyer-Olkin (KMO) measure of sampling adequacy was over 0.8. However, results showed less consistency between statements on economic issues, with a scale reliability coefficient of only 0.55 and a KMO value of 0.63. The higher degree of uniqueness among these responses suggests that there is no single, coherent left-right spectrum even among views on related topics, all touching on government spending and the economy; thus, it is unlikely that there are high levels of ideological polarization on these issues. Because economic issues include those which clearly and directly affect individual participants (e.g., changes to taxation or social welfare policies) as well as more abstract ideological (e.g., on the role of government) or technical (e.g., national debt) questions, it is not surprising that many people had varied answers based on their knowledge, beliefs, and experiences. By contrast, these findings highlight that there is consistency on socio-cultural issues, which suggests more decided, coherent views on topics of racial diversity and immigration. These findings justify assigning overall scores to participants based on their responses to socio-cultural statements; for the economic statements, results are borderline as to their suitability for being treated as a single score. We have opted to do so because we believe that reporting on the overall relationship between views on economic and socio-cultural issues is valuable; furthermore, using overall scores still identifies which participants had consistent, polarized responses, and such categories prove highly illustrative in our subsequent analysis.

In addition to the main latent factor, a secondary factor was also present in both sets of statements, which was driven by the items that had been inverted and were originally worded to favor the conservative stance. This secondary factor pointed to an evident bias among many participants towards agreement with whatever statement was provided, regardless of its ideological leaning. Given that more questions were worded to favor the progressive stance, we therefore opted to calculate overall scores using averages that weighted the conservative-leaning statements more heavily, offsetting the effects of this bias. Such weighting offers a more generous calibration to show potential polarization, and it serves as a robustness check against people’s hesitation to answer extreme response options. For the parts of our analysis that rely on these scores, our sample includes the 895 participants who fully answered the questions used to create our indexes.

To examine partisanship, we look at measures of party support and, separately, participants’ voting history and intentions. Beginning with support, we asked whether participants supported a particular political party or parties and, if so, which party or parties. For those who did not claim to support any particular party, we looked at information about who participants voted for in the previous election and who they planned to vote for in the next election to get clues about their party leanings. Differences in clustering among party supporters compared to voters who do not support a party should reveal the degree to which partisans are actually polarizing, rather than merely self-sorting. Because responses were open-ended, levels of support for federal versus provincial political parties cannot be determined. Only a handful of participants specified at which levels they supported a given party, and very few of these cited differences in their support between these levels.

Our sample, however, allows us to examine the differences between those who support the Liberals, Conservatives (or Progressive Conservatives), and, combined, two smaller parties, the New Democratic Party (NDP), and the Green Party, which are usually considered to be centre-left on the political spectrum. While the NDP and Greens each had a small number of unique supporters in our sample, it was also common for supporters of these parties to support multiple left-leaning parties; many supported at least two and sometimes all three of the NDP, Liberals, and Greens. This can be attributed partly to strategic motivations among the supporters of smaller parties (for example, they may vote “ABC,” i.e., Anything but Conservative), though it may also suggest an increasingly coherent and popular ideology forming on the political left in Canada (see Cochrane, 2010). Because such choices were common and, together, represent a larger group of mixed centre-left party supports, we combine these supporters into a single category. On the political right, most named the Conservatives or PCs, but a few also supported the People’s Party of Canada (PPC) or People’s Alliance of New Brunswick. To capture this small number of people who indicated support for the PPC or People’s Alliance, we group them together as right-wing populist party supporters, including those who expressed support for both the Conservatives and either the PPC or People’s Alliance. Other participants supported different parties (minor or foreign), or they supported multiple parties that crossed the left-right divide, e.g., both Liberals and Conservatives. These combinations occurred infrequently and are excluded from the analysis of partisan sorting and partisan polarization.

Thematic coding of open-ended responses was done by the authors with an interest in determining whether participants are perceiving increased polarization and, more generally, what kinds of political changes they are observing. Prior to being asked specific questions about their political views, participants were asked open-ended questions about the biggest changes they had observed in Canadians’ political activities and political views in the last few years. They were first asked, “If you have noticed any changes in the political activity of Canadians compared to 5 years ago, could you please explain the biggest changes you have noticed?” If they answered affirmatively to a question about whether it seems that Canadians’ political views have changed at all compared to 5 years ago, they were then asked, “Could you please explain the biggest changes you have noticed in the political views or beliefs of Canadians compared to 5 years ago?” Responses to the two questions were analyzed together because many participants conflated the concepts of political activity and political views, discussing changes in views in response to the question about political activity and vice versa; the answers they provided demand the more holistic concept of political change, which incorporates changes in political activity as well as in views, attitudes, and identities.

Telephone interviewers were instructed to record participants’ full responses to open-ended questions and repeat these recorded responses back to participants to confirm their accuracy. In total, 870 participants gave substantive responses. These responses ranged from a single word to 97, with a mean length of 32 words. Codes were generated inductively to reflect the relative frequency of “polarization” themes compared to others and to represent participants’ views and perceptions more holistically. Many participants raised multiple themes; the mean number of themes mentioned by each participant was 2.8.

This part of the analysis begins with descriptive reporting of participants’ perceptions, focusing especially on themes that relate to political polarization. We then use Multiple Correspondence Analysis (MCA) to look at these perceptions alongside participants’ views on socio-cultural and economic issues in order to examine the relationship between more extreme or polarized views and perceptions of polarization. Unlike regression, an MCA plot maps underlying patterns of responses, revealing clusters that can offer insight into the social spaces in which polarized views and perceptions of polarization are occurring. Whether or not there is evidence of ideological or partisan polarization, the clusters mapped by MCA can reveal who is perceiving ideological or affective divides, and they may also point to underlying patterns that help us to better understand these perceptions. In this analysis, views scores for each index are sorted into categories: 1) “very conservative” for scores of 2 or less, 2) “conservative” for scores from 2 to 3, 3) “progressive” for scores from 3 to 4, and 4) “very progressive” for scores over 4. Because of the important role social media plays in contemporary polarization and political discourse, we include two dummy variables in this analysis: one represents whether or not participants have a social media account, while the other represents whether or not participants engage in online discussions of political issues. Participants were asked how frequently they engage in online discussions of social or political issues; those who responded “sometimes” or “often” are considered to engage in these discussions, while those who answered “never” or “rarely” are not.

## Analysis

**FIGURE 1:**
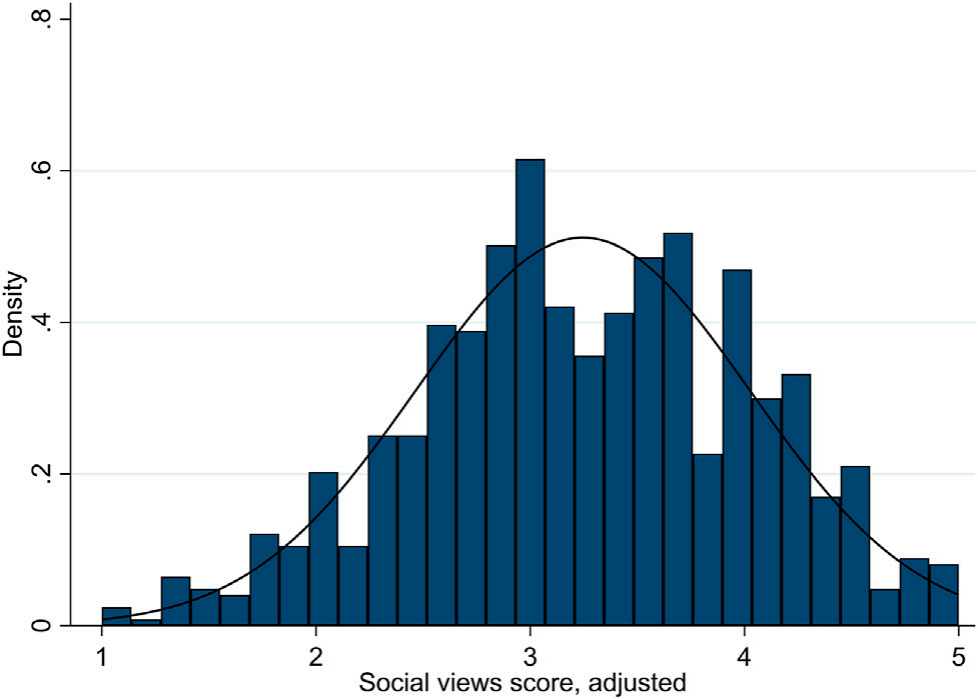
Distributions of views scores on socio-cultural issues.

**FIGURE 2:**
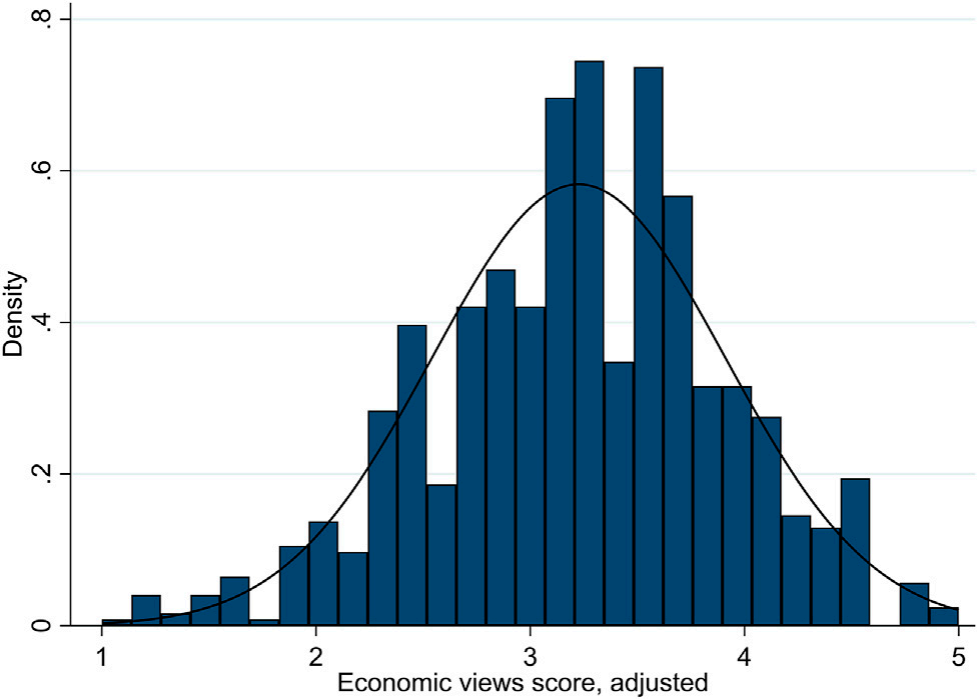
Distributions of views scores on economic issues.

We begin by looking at participants’ views on socio-cultural and economic issues to determine whether there is any evidence that ideological polarization is occurring. After accounting for participants’ bias towards agreement with the statements as presented, we find the mean adjusted score on the scale from 1 to 5 was 3.29 on socio-cultural issues and 3.23 on economic issues, with median scores of 3.35 and 3.25, respectively. (Without adjusting for bias, mean scores would be higher: 3.51 and 3.35, respectively.) Instead of the bimodal distribution with clustering at the poles that would signal ideological polarization, the scores are approximately normally distributed, but slightly left skewed. Figures 1, 2 show these distributions.

These results do not indicate a high level of ideological polarization; rather, the majority of participants appear to have fairly centrist, uncertain/mixed, or only somewhat progressive-leaning views. Only a minority of participants had scores which placed them towards the poles: about 19.4% of participants had scores of 4 or higher on socio-cultural issues (“very progressive”), while only 6.6% had scores of 2 or lower (“very conservative”). On economic issues, about 14.1% had scores of 4 or higher, and only 4.7% had scores of two or lower. The greater prevalence of very progressive views compared to very conservative views on diversity aligns with many recent polls of the region (see, e.g., EKOS Politics, 2019). (For additional information on the distribution of views by demographic categories, see Table 2A in the Supplementary Appendix.) We also find no evidence that the socio-cultural and economic dimensions are flattening into a single dimension of politics. The Pearson correlation coefficient is only 0.32 between the two sets of scores; thus, scores in each index are evidently correlated, but modestly.

**FIGURE 3:**
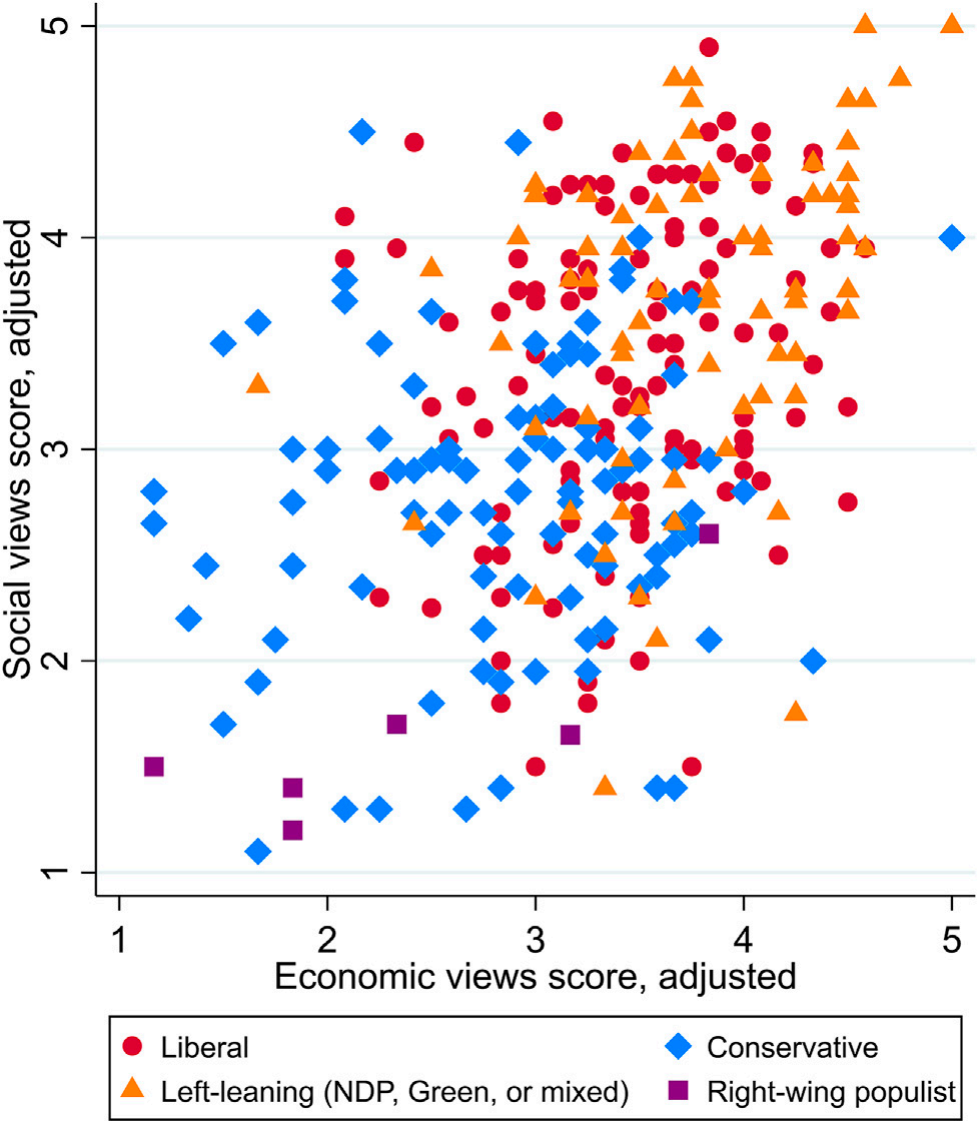
Views scores on socio-cultural and economic issues, by party supporters.

Turning to the analysis of partisan sorting and partisan polarization, we find, first of all, that 393 participants (43.9%) say they support a particular party or parties. Among these participants, 132 supported the Liberals, 99 supported the Conservatives, 76 supported one or more of the left-leaning parties, and only 6 supported right-wing populist parties. Another 80 participants supported other parties (minor or foreign), supported parties on both the left and the right, or refused to specify. Looking at the differences in scores between party supporters, shown in Figure 3, it is clear that parties occupy somewhat different but highly overlapping “spaces” when it comes to their views on socio-cultural and economic issues. The views of Liberal supporters are widely dispersed, but they tend to be grouped closer to the top right of the chart, indicating more progressive views. Conservative supporters, on the other hand, tend to be grouped somewhat closer to the bottom left, indicating more conservative views on both socio-cultural and economic issues; however, there is no tight clustering, and the dispersion of views at different points across both dimensions shows no hint at increasing consensus even within the single party. Because there are differences between the party platforms and governing ideologies, some correlation between views and party support is to be expected as a result of the partisan sorting process. The lack of tight clustering, however, suggests that partisan sorting is not strong in Atlantic Canada around socio-cultural and economic issues. In other words, people united in supporting a specific party are still far from being united in their views in these dimensions, while supporters of different parties may yet have very similar views.

The supporters of left-of-centre parties largely occupy the space toward the top right, in the same area as many Liberal supporters, although a handful of these points occur closer to the bottom right, indicating more conservative views on socio-cultural issues. The small handful of right-wing populist party supporters were unanimous in their ultra-conservative views on socio-cultural issues, although they differed on economic issues. These results are limited, however, by a small sample size. Few participants reported supporting these parties, but this reflects the general lack of support for the PPC in Atlantic Canada and small pockets of support for the People’s Alliance in New Brunswick.

**FIGURE 4:**
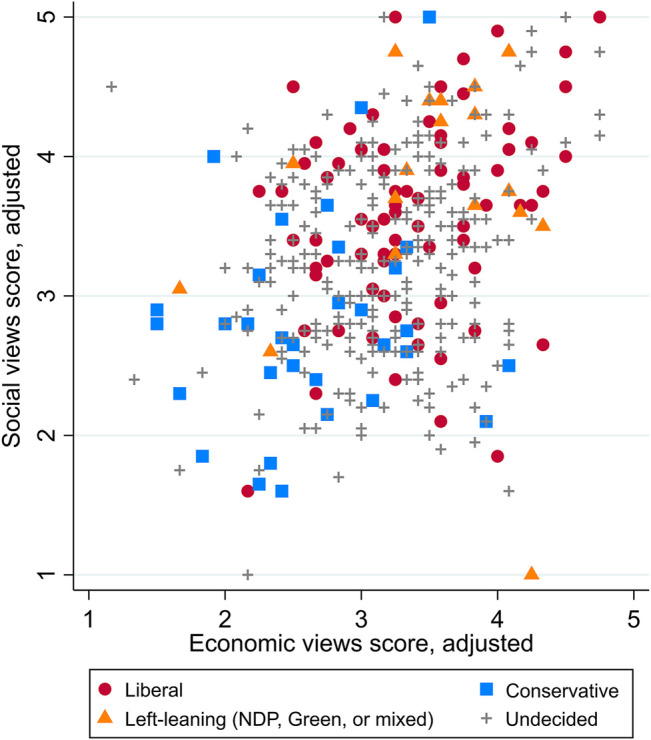
Views scores on socio-cultural and economic issues, by vote choice (excluding party supporters).

Looking at the vote choices of participants who do not support any party in Figure 4, patterns in socio-cultural and economic views are similar to those of partisans shown in Figure 3. Thus, self-identification as a party supporter does not appear to be linked to any additional clustering. Regardless of whether Atlantic Canadians identify themselves as supporting a specific party, they show similar variety in their political views across socio-cultural and economic dimensions, and their vote choices reflect similar sorting processes. Moreover, of the many participants who do not support a specific party, 267 people, or about half—with views from all over the spectrum on socio-cultural and economic issues—claimed to be undecided as to their voting intentions (another 27.4% refused to say). These findings do not point to signs of any significant degree of partisan polarization in the region beyond the limited effects of sorting processes. Rather than observing clear ideological differences between the parties and choosing one or another to support, Atlantic Canadians have mixed views and many lack the clear party loyalties that would drive partisan polarization.

### Perceptions of Polarization

Despite the lack of evidence for ideological polarization, or even for a high degree of partisan sorting, some participants noted their concerns over increased polarization and “more extreme” views when we asked about the biggest changes in political activities and political views they had observed among Canadians in the last 5 years. Among all survey participants, 870 people gave substantive responses to these questions. The most common themes and their frequencies are shown in Table 1. Coding open-ended responses, we found that 75 participants, or 8.6%, mentioned increased polarization as one of the biggest changes; this figure includes those who used the term “polarization,” as well as those who mentioned people getting further apart on the political spectrum or more divided into opposing political camps. Another 56, or 6.4%, mentioned that Canadians had stronger, more entrenched or more extreme views, or that they were becoming less open-minded. These responses did not necessarily point specifically to “two sides” or distance between ideological groups, but they correspond to affective polarization and, more generally, to the changes in the tone and tenor of political discourse that are raising alarm bells. Relatedly, 80 participants, 9.2%, mentioned that Canadians were becoming less tolerant or they noted the rise of far-right views or right-wing populism in Canada. Many noted this with evident negative judgments, citing concerns about racism and xenophobia, while some others expressed sympathy with what they considered a growing frustration among Canadians: by accepting high numbers of refugees and immigrants, the government is prioritizing “others” over “our own.” Immigrants and refugees to Canada were mentioned by 97 participants, or 11.1%, as a major change with significant political consequences. The increasing influence of U.S. politics in Canada—including the so-called “Donald Trump effect”—in Canada was mentioned by 105 participants, or 12.1%; this “Americanization” of Canadian politics was typically mentioned with a negative judgment.

**TABLE 1:** Most commonly perceived political changes by Atlantic Canadians.

Themes	Examples of common terms, phrases, and ideas	Count	%
Negativity	Disenchantment, losing faith, outraged, given up hope	260	29.9
Discussing politics	More talking, more discussions, more vocal	252	29.0
Social media and the internet	Facebook posts, online comments	152	17.5
More participation	More involved, more activities, etc.	145	16.7
Age and generational differences	Millennials, young people, generation	115	13.2
The United States	Trump, attention to/influence of U.S.	105	12.1
Immigration	Refugees, newcomers, immigrants	97	11.1
More protesting	More rallies, demonstrations, social movements	92	10.6
More knowledge or awareness	Paying more attention, more informed	83	9.5
Liberal Party of Canada	Justin Trudeau, the Liberals	80	9.2
Intolerance	Anti-immigrant, racism, far-right, populism	80	9.2
Environment	Climate change, pipelines, more green	80	9.2
Polarization	More polarized, more divided, farther right and farther left	75	8.6
Interest in politics	More interested, more concerned, care more	64	7.4
Voting less	Voter turnout is down, people don’t bother voting	63	7.2
Stronger, more entrenched views	More entrenched in opinions, more closed-minded	56	6.4

Polarization and related issues, while common, were not the most common themes raised by participants. However, the most common themes were frequently linked to polarization by participants who gave more detailed responses. The most common theme, for example, with 260 mentions or 29.9%, was negativity or dissatisfaction with the status quo. This was mentioned by participants with evident leanings to the right and, to a lesser extent, the left; for some, it was linked to increasing participation (e.g., “fighting back,” “standing up for ourselves”) and, for others, it was linked to apathy (e.g., “it doesn’t matter what we do, nothing changes”). The media, and especially social media, were implicated by many participants as playing a causal role in many of these changes: 152 participants, or 17.5%, mentioned the internet or social media one of the biggest changes in Canadian politics, affecting people’s views, political activities, and the broader discourse, including heightening tensions. The second most common theme mentioned was the increase in the number of people talking about politics or the frequency of political discussions, with 252 mentions or 29.0%; these discussions were often explicitly attributed by participants to social media or other internet forums. Based on the work of Baldassari and Bearman (2007), we would expect such increased exposure to the political opinions of friends and family to increase people’s awareness of political disagreements, thereby increasing perceived polarization.

**FIGURE 5:**
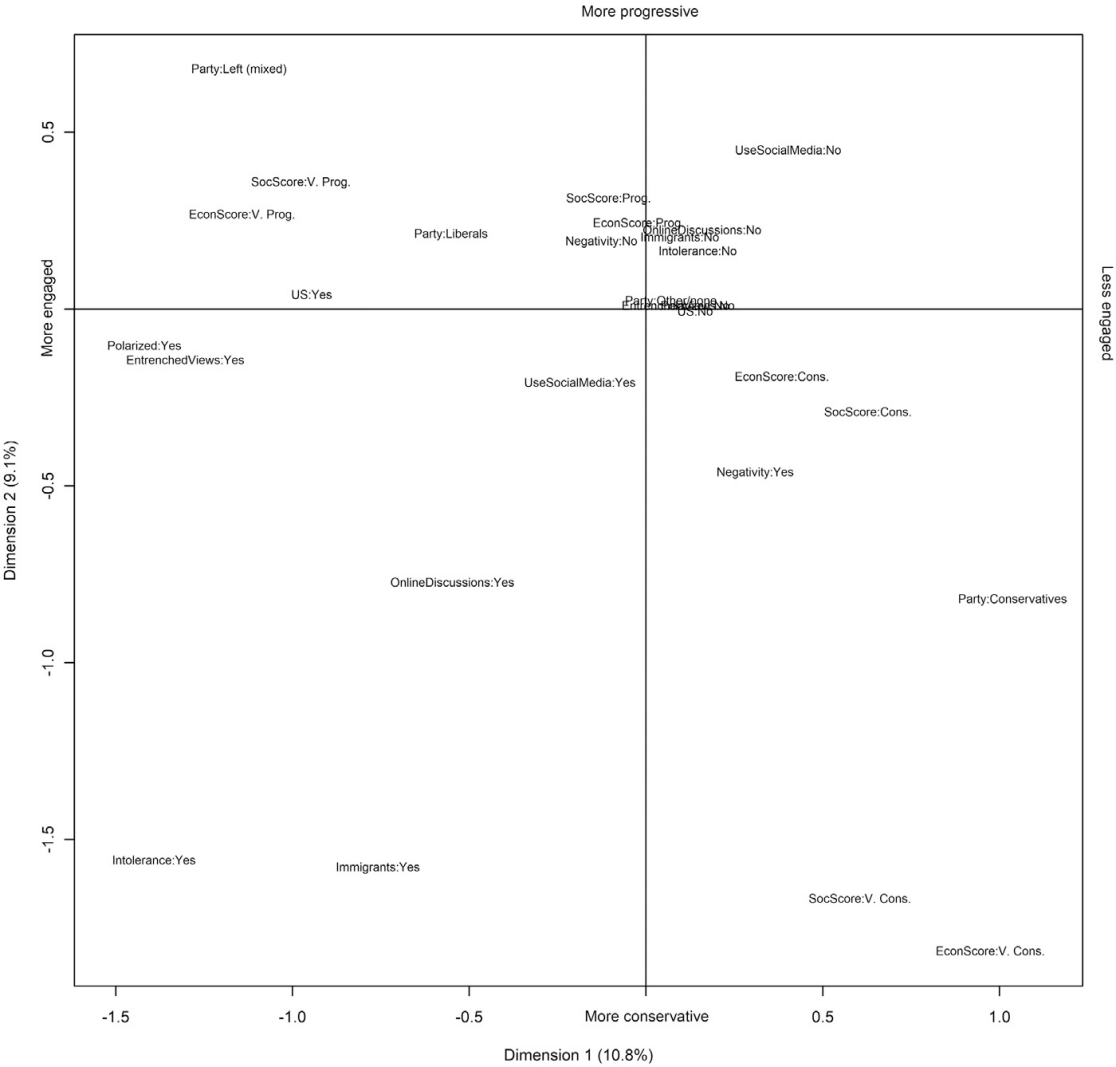
Multiple correspondence analysis of perceptions, political views, partisanship, and online engagement.

To examine the connections between these perceptions of polarization and division and participants’ partisanship and views on socio-cultural and economic issues—that is, to understand who is seeing what—we use Multiple Correspondence Analysis to “map” these relations (Figure 5). In addition to perceptions and views, we include dummy variables indicating whether participants have social media accounts or reported discussing political or social issues online in order to see how online engagement may be linked to certain kinds of views or perceptions of polarization. The clustering near the origin point can be understood as Atlantic Canadians’ politics-as-usual, as these categories are typically common and/or undifferentiated. This space also represents those who did not mention polarization, more extreme views, or other related perceptions, as well as those who do not discuss political issues online.

The vertical axis of the map corresponds to participants’ political views, running top-to-bottom from progressive to conservative. Accordingly, a cluster of “very conservative” scores on socio-cultural and economic issues is shown at the bottom-right of the map. This cluster reveals both the infrequency and high degree of differentiation of “very conservative” views on socio-cultural and economic issues among participants, and their close relationship to each other. While no particular perceptions of change were strongly and uniquely linked to this cluster, mentioning negativity or dissatisfaction is clearly related to holding these views—but this perception was also common among those classified as “conservative,” as well as those who support the Conservatives. To the extent that this negativity may be increasingly vocalized, this change could be contributing to affective polarization and perceptions of polarization in the region.

The “very progressive” cluster is found in the top-left quadrant of the map, between Liberal Party of Canada and mixed left-leaning party supporters. It is less distant from the origin point, at least along the vertical axis. Corresponding more closely with perceptions of greater polarization, more extremism/less open-mindedness, and the influence of U.S. politics in Canada, this cluster is more highly differentiated from Atlantic Canadians’ politics-as-usual by its engagement with and awareness of contentious political issues than by its political views, which are only slightly farther left than the norm.

The horizontal axis corresponds to engagement with polarizing issues, with the highly engaged situated on the left side of the map. The bottom-left quadrant, therefore, suggests there is a shared space of engagement between the engaged left-wing, located at the top-left of the map, and the distinct cluster of those with “very conservative” views at the bottom-right. The variables in this quadrant include perceptions of change due to immigration and perceptions of decreasing tolerance among Canadians; these perceptions are evidently linked to each other, and primarily associated with right-wing views. On the other hand, perceptions of polarization and the entrenchment of views are more common among those with more left-leaning views, but they are nonetheless within this shared space of engagement and debate. This quadrant also includes engagement with online political discussions; simply using social media, on the other hand, is located much closer to the origin.

From this mapping, we conclude that, while neither political polarization nor perceptions of polarization are prominent in Atlantic Canada, small clusters of people with “polarized” viewpoints and perceptions can easily be found in the region, and it is those people who most often see signs of polarization. While Atlantic Canadians display, on average, centre-to-centre-left views on both socio-cultural and economic views, some residents—with either left- or right-wing views—are engaging with polarizing issues and are highly aware of the potential effects of U.S. politics and/or polarizing rhetoric around immigration and tolerance in Atlantic Canada. Many are actively engaging with these ideas in online spaces, although we cannot determine from this evidence whether such engagement precedes or is a consequence of polarization processes. Nevertheless, it is clear that, in Atlantic Canada, engagement with polarization narratives is related to one’s own alignment with either very progressive or very conservative views. These two clusters have very different feelings and attitudes in response to polarization narratives, but they debate common issues.

## Conclusion

Polarization may be a global trend, but it manifests differently, and to varying degrees, across regions. In this paper, we set out to determine if there is evidence of ideological and partisan polarization in Atlantic Canada, an understudied, peripheral region. We also looked for perceived polarization and sought to understand the underlying patterns associated with those perceptions. Despite frequent concerns about political polarization in Canada, we find no evidence of mass ideological or partisan polarization in Atlantic Canada. Indeed, such polarization is rare and unlikely, although other forms—including elite-level polarization, partisan sorting, and affective polarization—may be more prevalent. The existence of extreme views and polarizing discourse in Canada, including Atlantic Canada, is undeniable; however, the majority of Atlantic Canadians are not ideologically polarized, even as social media makes differences more visible, amplifying perceptions of polarization and shaping popular concerns around it.

Although we do not find pervasive ideological and partisan divisions in the region, we do find evidence of increasing ideological similarity among many supporters of centre-left parties in Atlantic Canada. Conservatives, meanwhile, tend to occupy a diffuse political space to the right. This is in line with findings by Cochrane (2010) and Johnston (2019), which point to greater ideological clustering on the left, as well as increasing affective distance between the Conservatives and “everybody else.” But, in Atlantic Canada, substantial overlap between the two remains, as well as many people who do not support a party and could vote either way. Therefore, while some ideological variation and partisan sorting are evident, the existing levels of clustering do not point to substantial partisan polarization on socio-cultural or economic issues.

Finally, despite the relatively progressive consensus in Atlantic Canada, there is a small, engaged minority perceiving increasing polarization and extremism. These individuals tend to hold very progressive views and are often highly engaged with concerns about the broader political climate outside the Atlantic region. At the other end of the spectrum, a tiny proportion hold very conservative views, and they may also be highly engaged on contentious matters and in polarizing debates, especially on immigration and tolerance for socio-cultural diversity in Canada. While research has shown that social media use can create “echo chambers” that increase polarization and skew perceptions, our findings suggest that perceptions of increasing polarization are primarily occurring among the smaller subset of engaged individuals who discuss political issues online. Thus, despite the absence of mass ideological polarization, for the relatively small number at the “extremes,” divergent perceptions and perceptions of divergence, aggravated by social media and online echo chambers, may prove increasingly difficult to reconcile.

## Data Availability

The raw data supporting the conclusion of this article will be made available by the authors, without undue reservation.
