# Supplementary material for: Do Polarization Narratives Apply to Politics on the Periphery? The Case of Atlantic Canada
Source: Front Sociol. 2021 Oct 22;6:655880. doi: 10.3389/fsoc.2021.655880 (PMC8570129; doi:10.3389/fsoc.2021.655880)
Supplement: Supplementary file 1 [file DataSheet1.docx]

**Appendix**

Table 1A: Statements used to create socio-cultural and economic scores

| Socio-cultural | Multiculturalism is an important part of Canadian society. |
| --- | --- |
|  | It is important for Canadian society to be open to diverse lifestyles and forms of self-expression. |
|  | The country would benefit from having more members of minority groups in positions of power. |
|  | It is important that Canada allows entry for all asylum seekers fleeing violence or persecution. |
|  | Immigrants bring vital skills and resources that benefit the Canadian economy. |
|  | Immigrants need to do more to integrate into Canadian society. |
|  | It is important that the federal government puts the needs of Canadian citizens who were born in Canada first in policy decisions. |
| Economic | A balanced budget should be a top priority for the Canadian federal government. |
|  | I would support raising taxes to expand or improve government services such as health care and education. |
|  | When services are provided by private businesses instead of by the government, they are usually more efficient and less expensive. |
|  | The minimum wage should be raised substantially to ensure that people who are employed are able to make ends meet. |
|  | It is the responsibility of the government to ensure that everyone in Canada has access to essentials, including things like food, shelter, heat, and health care. |

Table 2A: Views scores on socio-cultural and economic issues in categories, by demographics

| Demographic categories | Socio-cultural issues | | | Economic issues | | | N |
| --- | --- | --- | --- | --- | --- | --- | --- |
|  | Very cons. | Neither | Very prog. | Very cons. | Neither | Very prog. |  |
| **Education** |  |  |  |  |  |  |  |
| High school diploma or lower | 8.8% | 80.9% | 10.4% | 4.4% | 83.3% | 12.4% | 251 |
| College or trade certificate, diploma, or degree | 10.8% | 75.4% | 13.8% | 3.9% | 88.8% | 7.3% | 232 |
| Undergraduate degree or higher | 3.0% | 68.4% | 28.6% | 5.5% | 75.9% | 18.7% | 402 |
|  |  |  |  |  |  |  |  |
| **Age group** |  |  |  |  |  |  |  |
| 18-34 | --* | 60.6% | --* | --* | 85.9% | --* | 71 |
| 35-49 | 6.9% | 72.8% | 20.2% | 7.5% | 82.7% | 9.8% | 173 |
| 50-64 | 7.7% | 72.5% | 19.8% | 4.6% | 82.7% | 12.7% | 323 |
| 65+ | 5.9% | 78.8% | 15.3% | 2.9% | 77.2% | 19.9% | 307 |
|  |  |  |  |  |  |  |  |
| **Gender** |  |  |  |  |  |  |  |
| Men | 9.1% | 75.0% | 15.9% | 7.5% | 78.1% | 14.4% | 416 |
| Women | 4.4% | 73.2% | 22.4% | 2.3% | 83.9% | 13.8% | 477 |
|  |  |  |  |  |  |  |  |
| **Province** |  |  |  |  |  |  |  |
| New Brunswick | 7.4% | 77.9% | 14.7% | 6.6% | 83.3% | 10.1% | 258 |
| Newfoundland & Labrador | 8.4% | 74.8% | 16.8% | 4.2% | 86.0% | 9.8% | 143 |
| Nova Scotia | 5.5% | 71.5% | 23.0% | 3.5% | 78.4% | 18.2% | 435 |
| Prince Edward Island | --* | 72.9% | --* | --* | 81.4% | --* | 59 |
| *Some results are withheld due to small sample sizes in the “very conservative” category. | | | | | | | |
